# Time course and predictive factors for lung volume reduction following stereotactic ablative radiotherapy (SABR) of lung tumors

**DOI:** 10.1186/s13014-016-0616-8

**Published:** 2016-03-15

**Authors:** Michael S. Binkley, Joseph B. Shrager, Aadel Chaudhuri, Rita Popat, Peter G. Maxim, David Benjamin Shultz, Maximilian Diehn, Billy W. Loo

**Affiliations:** Department of Radiation Oncology, Stanford University School of Medicine, 875 Blake Wilbur Drive, Stanford, CA 94305 USA; Stanford Cancer Institute, Stanford University School of Medicine, 875 Blake Wilbur Drive, Stanford, CA 94305 USA; Institute for Stem Cell Biology & Regenerative Medicine, Stanford University School of Medicine, 875 Blake Wilbur Drive, Stanford, CA 94305 USA; Department of Cardiothoracic Surgery, Division of Thoracic Surgery, Stanford University School of Medicine, Stanford, USA; Department of Health Research & Policy, Stanford University School of Medicine, Stanford, USA; Princess Margaret Cancer Centre, Toronto, Ontario Canada; University of Toronto, Toronto, Canada

**Keywords:** Stereotactic ablative radiotherapy, Emphysema

## Abstract

**Background:**

Stereotactic ablative volume reduction (SAVR) is a potential alternative to lung-volume reduction surgery in patients with severe emphysema and excessive surgical risk. Having previously observed a dose-volume response for localized lobar volume reduction after stereotactic ablative radiotherapy (SABR) for lung tumors, we investigated the time course and factors associated with volume reduction.

**Methods:**

We retrospectively identified 70 eligible patients receiving lung tumor SABR during 2007-2013. We correlated lobar volume reduction (relative to total, bilateral lung volume [TLV]) with volume receiving high biologically effective doses (V_XXBED3_) and other pre-treatment factors in all patients, and measured the time course of volume changes on 3-month interval CT scans in patients with large V_60BED3_ (*n* = 21, V_60BED3_ ≥4.1 % TLV).

**Results:**

Median CT follow-up was 15 months. Median volume reduction of treated lobes was 4.5 % of TLV (range 0.01–13.0 %), or ~9 % of ipsilateral lung volume (ILV); median expansion of non-target adjacent lobes was 2.2 % TLV (−4.6–9.9 %; ~4 % ILV). Treated lobe volume reduction was significantly greater with larger V_XXBED3_ (XX = 20–100 Gy, *R*^*2*^ = 0.52–0.55, *p* < 0.0001) and smaller with lower pre-treatment FEV1% (*R*^*2*^ = 0.11, *p* = 0.005) in a multivariable linear model. Maximum volume reduction was reached by ~12 months and persisted.

**Conclusions:**

We identified a multivariable model for lobar volume reduction after SABR incorporating dose-volume and pre-treatment FEV1% and characterized its time course.

**Electronic supplementary material:**

The online version of this article (doi:10.1186/s13014-016-0616-8) contains supplementary material, which is available to authorized users.

## Background

Emphysema, a subtype of chronic obstructive pulmonary disease (COPD), is a progressive disease with few available interventions providing long-term benefits. As demonstrated by the National Emphysema Treatment Trial (NETT), lung-volume-reduction surgery (LVRS) improved quality of life, exercise tolerance, pulmonary function in select patients, and increased survival for a subset with low exercise capacity and upper lobe-predominant, heterogeneous disease [1]. Additionally, up to 15 % of patients with advanced emphysema may be suitable to undergo LVRS [2]. However, many patients with severe emphysema are not surgical candidates, as they may have excessive comorbidities or exhibit features associated with a striking 16 % 90-day mortality rate in the NETT and are classified as high-risk [1, 3]. Importantly, at 6-year follow up of 140 high-risk NETT patients, those receiving LVRS demonstrated equivalent long-term overall survival with significantly improved quality of life compared with those medically managed [4]. Thus, investigation of less invasive LVRS alternatives for lung volume reduction is warranted for high-risk emphysema patients.

Stereotactic ablative radiotherapy (SABR) delivers highly conformal and ablative doses of radiation, achieving high rates of local control, disease-free survival, and overall survival in patients with clinical stage I non-small cell lung cancer [5]. In animal models, histopathologic analysis after stereotactic radiation demonstrates parenchymal fibrotic scarring, fibrosis of involved pleura, alveolar air space narrowing, alveolar septal thickening, and intimal thickening of arteries [6]. In patients with lung tumors, computed-tomography (CT)-based assessments of post-SABR parenchymal changes have predominantly investigated fibrosis volume and density changes [7–10]. Chronologic measurements of lung fibrosis after SABR demonstrate stable fibrosis volume approximately 18 months post-treatment [11].

Having observed in our practices lung parenchymal scarring and tissue contraction following lung tumor SABR, we hypothesized that SABR might, as a secondary effect of tumor treatment, achieve volume reduction (stereotactic ablative volume reduction, (SAVR)). If we can establish that this in fact occurs, then our longer-term goal will be to develop this into a potentially less morbid means of creating therapeutic lung volume reduction in emphysema patients.

We previously identified a linear dose-volume response between treated lobe volume reduction and lobar volume receiving biologically effective dose ≥60 Gy. We did not observe significant correlation between volume of fibrosis and volume reduction [12], which is not unexpected given that scars contract and the volume of the final scar may not correlate directly to the original volume of corresponding lung parenchyma. To further this line of study, we herein investigate the time course of volume changes following SABR for lung tumors and explore other pretreatment factors that might be predictive of volume reduction in a larger cohort.

## Methods

### Patients

We conducted a retrospective review, with Institutional Review Board approval, of patients receiving lung tumor SABR at our institution between 2007 and 2013 meeting our inclusion criteria: no prior thoracic radiation treatment, pretreatment ^18^FDG-PET/CT, follow-up imaging by CT >6 months post-SABR, and no subsequent thoracic treatment during imaging follow-up.

### Treatment and follow-up

We have previously reported our treatment methods [13]. In brief, treatment covered the PTV with 95 % of the prescribed dose and placed the center point of the maximum dose, typically 120 % of prescribed, within the GTV. Patients in this cohort were treated using either CyberKnife with multiple non-planar beams (Accuray Inc., Sunnyvale, CA), or Trilogy or TrueBeam (Varian Medical Systems, Palo Alto, CA) with coplanar arc delivery with x-ray CT cone beam for anatomy-based matching with some treatments utilizing respiratory gating. Patients received 25–60 Gy (median 50) in 1–8 fractions (median 4). Following SABR, patients were routinely followed at 3-month intervals with diagnostic CT, PET/CT, or both for the first two years and as indicated afterwards.

### Toxicity

We graded esophageal injury, radiation pneumonitis, rib fracture, and chest wall toxicity using the National Cancer Institute Common Terminology Criteria for Adverse Events (NCI CTCAE) version 4.03.

### CT and ^18^FDG-PET imaging

Pretreatment diagnostic CT imaging within 6 months prior to treatment and including the entire lung field was available for a subset of patients. All patients received pretreatment PET/CT imaging. SABR treatment planning CT scans consisted of exhalation breath-hold (slice thickness, 1.25 mm) and free breathing 4D-CT scans (slice thickness, 2.5 mm). All pretreatment and post-treatment diagnostic CT imaging had slice thickness from 1 to 5 mm (median 1.25 mm, mode 1.25 mm).

### Contouring, dosimetric analysis, and standard uptake value

Lung lobes were manually contoured on axial CT slices on lung window setting (window = 1400 HU, level = -500 HU) by a single observer (MSB) using MIM software, version 6.1 (Cleveland, OH) and were verified by an experienced thoracic radiation oncologist (BWL). The right upper and middle lobes were contoured together when the dividing fissure was not well visualized. For patients with multiple tumors in separate lobes, the treated lobe volumes were summed, representing the treated volume. As we compared scans with different inhalation volumes, the lobar volumes were represented as relative volumes to the total lung volume (TLV). Baseline pretreatment lobe volume was obtained from diagnostic pretreatment CT scans for the majority of patients, but as surrogate for all others, pretreatment lobe volume was obtained from simulation treatment planning CT scans. We compared relative lobe volume for patients with both pretreatment diagnostic CT scans and treatment planning CT scans to validate our method. We also assessed visually the presence of post-treatment atelectasis distal to an ablated airway (manifest as wedge like pattern of density distal to airways) *vs.* parenchymal loss from fibrosis in the absence of atelectasis distal to an airway.

We measured V_XXBED3_, defined as the volume of the treated lobe receiving a biologically effective dose BED_3_ ≥ XX Gy (α/β = 3 for late effects, XX = 20, 40, 60, 80 and 100 Gy BED_3_) from the dose-volume histograms of the radiation treatment plans. The target volumes were not excluded from the V_XXBED3_. BED_3_ was calculated using the linear-quadratic (LQ) model.

Total lung and lobar volumes contoured on treatment planning CT scans were fused with pretreatment attenuation-corrected PET scans with subtraction of the PTV to define non-target lung tissue. The standard uptake value (SUV) for the mean, 85, 90, and 95^th^ percentile were measured for the non-target total lung volume, ipsilateral lung volume, and treated lobe volume. The maximum SUV for the GTV was also measured.

### Pulmonary function testing

The forced expiratory volume in one second as a percent of the predicted value (FEV1%) was extracted from pulmonary function testing (PFT).

### Statistical analysis

We compared the following variables with treated lobe volume reduction (as % of bilateral TLV) using univariate linear regression: pre-SABR FEV1%, V_XXBED3_, PTV (%TLV), and adjacent lobe expansion. We included variables that significantly correlated (alpha = 0.05) with treated lobe volume reduction in our multivariable linear regression model. For exploratory purposes, we compared treated lobe volume reduction and V_60BED3_ using multivariable linear regression testing for interaction between V_60BED3_ and the following pretreatment variables: FEV1%, number of fractions (single *vs.* multiple), follow-up time, proximity (central *vs.* peripheral), location (upper *vs.* lower), treated lobe volume <−910 HU [14], V_20BED3_-PTV ratio, V_20BED3_-shell (subtracting V_40BED3_ from V_20BED3_), mean SUV, SUV85, SUV90, SUV95, maximum GTV SUV, pre-SABR WBC count, absolute neutrophil count, neutrophil-lymphocyte ratio, and SABR delivery platform (CyberKnife using numerous non-coplanar beams *vs.* Trilogy/TrueBeam using coplanar arc-based IMRT).

For the 21 patients receiving the upper tertile of V_60BED3_ (V_60BED3_ ≥4.1 % TLV, representative of targets for the emphysema application) with interval diagnostic CT imaging available, we utilized a repeated-measure, mixed-model analysis to compare treated lobe volume reduction at interval CT scans for the same pretreatment variables noted above. Patients without diagnostic CT imaging at a given interval time point most frequently received solely PET/CT imaging. Fisher’s exact test was used to compare incidence of pneumonitis for (1) patients receiving V_60BED3_ ≥4.1 % TLV against all others, and (2) patients with GOLD COPD stage III/IV versus stage I/II.

Paired *t*-test was used to compare (1) pre- and post-SABR PFT, and (2) relative lobe volume measured on pretreatment diagnostic and treatment planning CT scans. All statistical analyses were performed using Stata software (version 13, College Station, TX). All interactions were tested at alpha = 0.05.

## Results

### Patient, tumor, and treatment characteristics

70 patients (28 men and 42 women) met inclusion criteria with characteristics summarized in Table 1. The most common factor for exclusion was subsequent thoracic radiation therapy. 68 had primary stage I non-small cell lung cancer and two had lung metastases from another primary site. Median age was 74 (range 54–91). Four patients had two tumors treated, and one patient had three tumors treated with the same course of SABR. Median duration of post-SABR CT follow-up was 15 months (range 8–26 months).

**Table 1:** Patient, tumor, and treatment characteristics

Parameter	Cohort (*n* = 70)
Patients-no.	70
Tumors (targets)-no.	76
Follow-up time-median (range)	15 (8–22) months
Median age-median (range)	74 (54–91) years
Sex	
M-no. (%)	28 (40.0)
F-no. (%)	42 (60.0)
Location	
Right upper lobe-no. (%)	18 (23.7)
Right middle lobe-no. (%)	4 (5.3)
Right lower lobe-no. (%)	14 (18.4)
Left upper lobe-no. (%)	14 (18.4)
Left lower lobe-no. (%)	26 (34.2)
Central	28 (36.8)
Peripheral	48 (63.2)
Metastatic-no. (%)	2 (2.9)
Lung Primary-no. (%)	68 (97.1)
Histology	
Adenocarcinoma	40 (57.1)
Squamous	17 (24.3)
NSCLC-NOS	11 (15.7)
Tumor Size-median (range)	2.3 (0.6–5.5) cm
≤3 cm	53 (69.7)
>3 cm	23 (30.3)
Median GTV (range)	6.6 (0.3–150.4) cc
Median PTV (range)	20.8 (12.4–308.2) cc
Median total dose (range)	50 (25–60) Gy
1 fraction (%)	19 (27.1 %)
3–8 fractions (%)	51 (72.9 %)

*Abbreviations: NSCLC* non-small-cell lung cancer, *NOS* Not otherwise specified, *GTV* gross tumor volume, *PTV* planning target volume

### Toxicity

During follow up, incidence of grade 1 and 2 pneumonitis was 7.1 and 10 %, respectively, with one case of grade 3 pneumonitis (1.4 %) in a patient with V_60BED3_ = 7.6 % of TLV (the ninth largest value). 21 patients received the upper tertile of V_60BED3_ (≥4.1 % of TLV). Incidence of grade ≥2 pneumonitis was significantly higher for these patients (*n* = 5 of 21, 23.8 %) versus all others (*n* = 3 of 49, 6.1 %, *p* = 0.047). Only 1 of 8 patients who experienced grade ≥2 radiation pneumonitis had pretreatment FEV1% <50 % predicted (*p* = 0.25, *n* = 1 of 21 for GOLD stage III/IV versus *n* = 7 of 43 for stage I/II).

For the entire cohort, incidence of grade 1 and 2 chest wall toxicity was 4.3 and 1.4 % respectively. Incidence of grade 1 and 2 esophagitis was 1.4 and 2.8 %, respectively. Incidence of rib fracture was 2.9 %.

### Lung volume changes

**Fig. 1:**
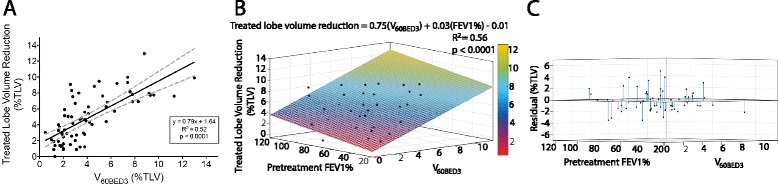
Treated lung volume change after SABR. **a** Treated lobe volume reduction (as % of total lung volume [TLV]) versus V_60BED3_ (% of TLV receiving a biologically effective dose ≥60 Gy). The significant linear correlation (*black*) indicates a dose-volume response relationship (95 % CI, *dashed-gray*). **b** Multivariable model (*n* = 64) comparing treated lobe volume reduction with V_60BED3_ and pretreatment FEV1%. **c** Residual plot of the same multivariable model demonstrating goodness of fit

Treated lobe volume reduction was associated predominantly with fibrosis and loss of lung parenchyma (*n* = 68), with only two examples associated with an atelectasis pattern. Median volume reduction of the treated lobe was 4.5 % of bilateral TLV (range 0.01–13.0 %), correlating significantly with the lobe volume receiving BED_3_ ≥ 20 (*R*^*2*^ = 0.51, beta = 0.5, 95 % CI = 0.38–0.61, *p* < 0.0001), 40 (*R*^*2*^ = 0.52, beta = 0.64, 95 % CI = 0.49–0.79, *p* < 0.0001), 60 (Fig. 1a, *R*^*2*^ = 0.52, beta = 0.8, 95 % CI = 0.62–0.98, *p* < 0.0001), 80 (*R*^*2*^ = 0.55, beta = 0.99, 95 % CI = 0.77–1.2, *p* < 0.0001), and 100 Gy (*R*^*2*^ = 0.55, beta = 1.18, 95 % CI = 0.92–1.44, *p* < 0.0001). Treated lobe volume reduction also correlated significantly with PTV (*R*^*2*^ = 0.31, beta = 184.5, 95 % CI = 119.8–249.3, *p* < 0.0001) and pretreatment FEV1% (*R*^*2*^ = 0.11, beta = 0.04, 95 % CI = 0.01–0.07, *p* = 0.005). A multivariable linear model was generated (Fig. 1b, *R*^*2*^ = 0.56, *p* < 0.0001):

$$ \mathrm{Volume}\ \mathrm{reduction} = 0.75\left({\mathrm{V}}_{60\mathrm{BED}3}\right) + 0.03\left(\mathrm{F}\mathrm{E}\mathrm{V}1\%\right)\ \hbox{--}\ 0.1 $$

**Fig. 2:**
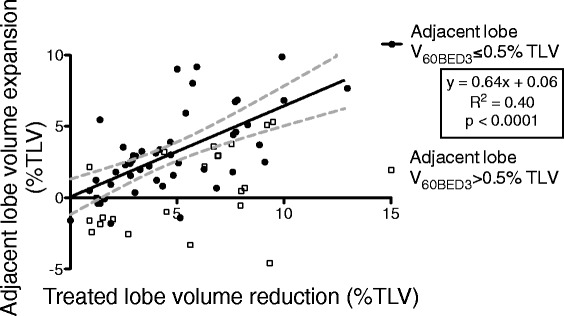
Adjacent lobe volume expansion *vs.* treated lobe volume reduction (as % of total lung volume). The significant correlation suggests compensatory expansion of the adjacent lobes after SABR, representing the desired effect of SAVR for emphysema. The linear correlation includes adjacent lobes receiving V_60BED3_ ≤0.5 % TLV (*n* = 48, *black dots*) and excludes adjacent lobes receiving V_60BED3_ ≥0.5 % TLV (*squares*), correcting for the treated volume extending across the lung fissure and encompassing adjacent lobe volume

Median expansion of the non-target adjacent lobe was 2.2 % of bilateral TLV (range −4.6–9.9 %). After observing treated volume extending across the lung fissure, we excluded adjacent lobes receiving V_60BED3_ >0.5 % TLV from our linear correlation comparing treated lobe reduction and adjacent lobe expansion (Fig. 2, *R*^*2*^ = 0.4, *p* < 0.0001). The median expansion of this subset was 2.5 % of TLV (range −1.8–9.86 %).

For 47 patients who had both deep inhale pre-SABR diagnostic CT and natural exhale pre-SABR planning CT, the relative volume of the treated lobe between the two breath-hold states was highly consistent with a mean difference of 0.1 % of TLV (95 % CI = −0.56–0.78 % of TLV, *p* = 0.77), supporting the method of analysis by relative volume and utilizing either pretreatment scan as baseline. When available, we utilized the pretreatment diagnostic relative lobe volumes as baseline (*n* = 47) and the exhale planning CT for the remainder (*n* = 23).

Exploratory analysis did not reveal statistically significant interaction between V_60BED3_ and the following variables in predicting target lobe volume reduction (Additional file 1: Table S1): pre-SABR FEV1% (*n* = 64), number of fractions (single *n* = 24 *vs.* multiple *n* = 46), follow-up time, proximity (central *vs.* peripheral), location (upper *vs.* lower), treated lobe volume <−910 HU, V_20BED3_-PTV ratio, V_20BED3_ shell (subtracting V_40BED3_ from V_20BED3_), median SUV, SUV85, SUV90, SUV95, GTV maximum SUV, pre-SABR WBC count, absolute neutrophil count (*n* = 58), neutrophil-lymphocyte ratio, and SABR delivery platform (CyberKnife, *n* = 10 *vs.* Trilogy/TrueBeam, *n* = 60).

### Time course of lobar volume changes post-SABR

**Fig. 3:**
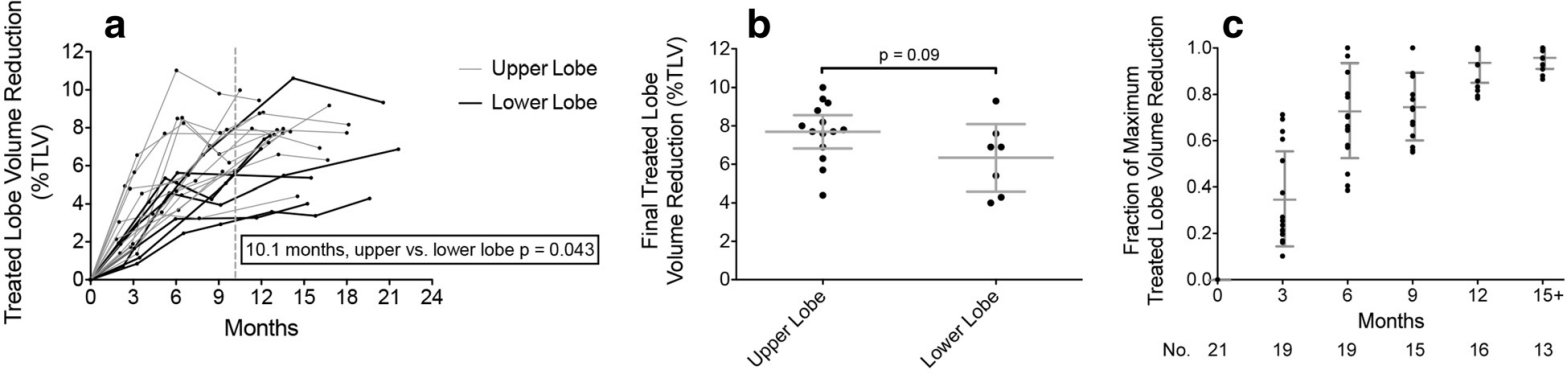
Time course of lung volume changes after SABR for upper tertile of V_60BED3_. **a** Treated lobe volume reduction (as % of total lung volume [TLV]) versus time (continuous) measured on serial diagnostic CT studies (*black dots*) and connected by a line for each patient (*n* = 21). The significant repeated-measure regression correlation indicates faster rate of volume change for upper (*gray*) versus lower (*black*) treated lobe location up to 10.1 months. **b** Treated lobe volume reduction measured on last CT follow-up indicating no significant difference in final volume reduction between upper and lower treated lobes. **c** Normalized (as fraction of maximum) treated lobe volume reduction (*black dots*) for each patient (*n* = 21) versus time (categorical) binned by 3-month intervals. Error bars demonstrate mean and 95 % confidence interval of the mean

**Fig. 4:**
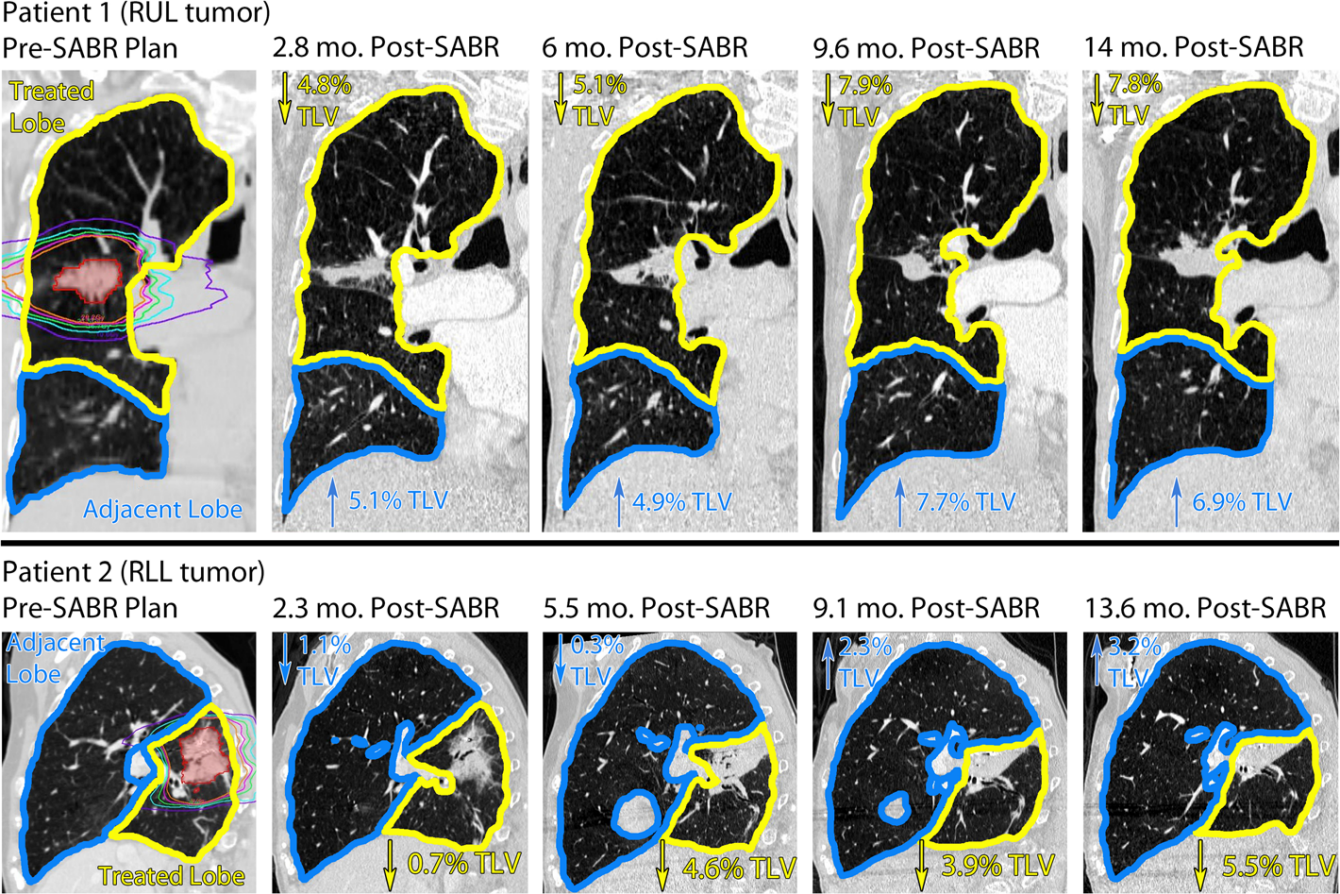
Example of lung volume changes over time after SABR to a right upper lobe tumor (Patient 1) and a right lower lobe tumor (Patient 2). Coronal (patient 1) and sagittal (patient 2) slices through the target volumes are shown. Gross tumor volumes are outlined in red and the 20 (*purple*), 40 (*blue*), 60 (*green*), 80 (*pink*), 100 Gy (*orange*) biologically effective dose (BED_3_) isodose volumes are outlined. Treated lobe volume (yellow) and adjacent lobe volume (*blue*) (both as % of total lung volume [TLV]) change relative to pretreatment volumes on serial diagnostic CT imaging, demonstrating progressive treated lobe volume reduction and adjacent lobe compensatory expansion

**Fig. 5:**
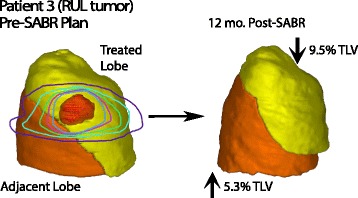
Three-Dimensional example of lung volume changes after SABR to a right upper lobe tumor (Patient 3). Gross tumor volume is outlined in red and the 20 (*blue*), 40 (*pink*), 60 (*green*), 80 (*teal*), 100 Gy (*purple*) biologically effective dose (BED3) isodose volumes are outlined. Treated lobe volume reduction (*yellow*) and adjacent lobe compensatory expansion (*orange*) (both as % of total lung volume [TLV]) is observed relative to pretreatment volumes on diagnostic CT imaging

We measured treated lobe volume reduction for 21 patients receiving V_60BED3_ ≥4.1 % TLV with available interval diagnostic CT imaging, observing a faster rate of volume reduction for treated upper lobes (*n* = 14) versus lower lobes (*n* = 7) up to 10.1 months of CT follow-up (Fig. 3a, upper lobe*time interaction, beta = 0.0023, 95 % CI = 0.00008–0.0045, *p* = 0.043). On final CT follow-up, there was no statistical difference between upper versus lower lobes (Fig. 3b, mean difference 1.35 % of TLV, 95 % CI = −0.24–2.93, *p* = 0.09). Furthermore, there was no significant interaction between upper lobe location and V_60BED3_ predicting the final treated lobe volume reduction for the entire cohort. For further normalization, we divided the change in treated lobe volume by the maximum observed change from pretreatment volume on follow-up diagnostic CT: 3-month (*n* = 19), 6-month (*n* = 19), 9-month (*n* = 15), 12-month (*n* = 16), and ≥15 month (*n* = 13) (Fig. 3c). Figure 4 demonstrates two patient examples of the lobar volume changes post-SABR. Figure 5 demonstrates a 3-dimensional visualization of lobar volume changes post-SABR.

### Pulmonary function testing

Sixty-four patients had pretreatment PFT available (median FEV1% 62.5, range 24–118) representing GOLD COPD stage I (*n* = 17), stage II (*n* = 26), stage III (*n* = 17), and stage IV (*n* = 4). 20 patients (*n* = 4, GOLD COPD stage III/IV) had pretreatment and post-treatment PFT available (median 12.2, range 3.1–29.5 months post-SABR). As anticipated, no significant difference was observed between pre- or post-SABR FEV1% (post-SABR FEV1% subtracted from pre-SABR FEV1%, mean 2.93, 95 % CI −2.41–8.27, *p* = 0.26) since tumors rather than areas of emphysema were targeted with SABR.

## Discussion

The goal of LVRS is to resect approximately 20 % of the ipsilateral lung volume (10 % of bilateral TLV) on each side. For bilateral LVRS, it has been established that the greatest benefit is for patients with upper lobe-predominant disease and low pretreatment exercise tolerance. Well-defined selection criteria and limitation of the procedure to experienced centers have reduced the LVRS mortality rate to <5 % [15]. Importantly, long-term follow-up of patients proven to be “high-risk” by the NETT, with 16 % 90-day mortality rate [2], demonstrates quality of life benefit in long-term survivors receiving LVRS [3], validating exploration of less invasive alternatives. A variety of bronchoscopic lung volume reduction approaches, including endobronchial valves [16], thermal ablation [17], and instillation of biologic agents [18], are under investigation though none are approved or generally available for clinical use [19]. Analogous to the adoption of SABR for medically inoperable early stage lung cancer, SAVR may offer such an alternative for patients with high surgical risk and severe emphysema, which while technically non-malignant, carries prognosis comparable to many lung cancers [20]. After previously identifying a dose–response relationship for treated lobe volume reduction following lung tumor SABR [12], we evaluated the time course of volume reduction and predictive pretreatment factors in a larger patient cohort.

Our findings verify a linear dose-volume response for V_XXBED3_ and treated lobe volume reduction in a 70-patient cohort, extending our previously reported dose–response curve to include larger SABR treatment volumes [12]. Patients receiving V_60BED3_ ≥4.1 % TLV achieved median treated lobe volume reduction of 7.7 % (range, 4–13 %, approximately 15.4 % of ILV), approaching the unilateral LVRS goal. Additionally, SABR treatment volumes occasionally encompassed lung tissue of the adjacent untreated lobe, likely producing further contraction. We corrected for this observation when analyzing the correlation between treated lobe volume reduction and adjacent lobe volume expansion and observed a ratio of approximately 3:2, comparable with volumetric studies following surgical resection of lung tissue [21].

Beyond dose, pretreatment FEV1% emerged as an independent variable that significantly associates with volume reduction, though with a beta-coefficient of lower magnitude than the dose-volume factor. We did not observe significant interaction between V_XXBED3_ and dose conformity, treated lobe volume with HU <−910, age, or parameters associated with inflammatory response such as WBC count and PET/CT SUV parameters of tumors and normal lung tissue.

Our findings suggest patients with severe emphysema and low predicted FEV1% achieve slightly smaller volume reduction for given V_60BED3_. This could be due to treatment volumes containing less tissue, and more air, in emphysematous versus non-emphysematous lung. This observation might seem to reduce the likelihood of successful volume reduction in patients most likely to benefit from SAVR. However, escalation of dose guided by our multivariable model may be feasible with acceptable toxicity. Lower rates of radiation pneumonitis are reported in patients with severe emphysema treated with SABR for stage I NSCLC [22]. Notably, only 1 of 8 patients developing grade ≥2 radiation pneumonitis in our cohort had pretreatment FEV1% <50 %, suggesting that patients with severe emphysema may be at lower risk of pneumonitis for a given treatment volume, allowing for dose intensification.

For patients receiving V_60BED3_ ≥4.1 % TLV (within the potential range for SAVR), we determined the time course of volume reduction. We observed faster rate of volume reduction for treated upper lobes versus lower lobes, but final follow-up imaging comparison suggests treated lower lobes ultimately achieve nearly equivalent volume reduction. This may represent one advantage for SAVR as an LVRS alternative, as resecting substantial portions of the lower lobes can be surgically challenging and has not consistently demonstrated comparable benefits to upper lobe LVRS. Additionally, 13 patients with long-term follow-up had persistence of volume reduction at ≥15 months, which is similar to the time course reported for stability of lung fibrosis at 18 months post-SABR [11].

Consistent with Stanic and colleagues [23], we did not observe significant change between pre- and post-SABR PFT. We note that this is expected in the patient population of the current analysis since treatment of lung tumors does not preferentially target lung parenchyma with hyper-expansion, as would be the goal of SAVR. Therefore lack of PFT changes in this setting is not informative regarding the potential emphysema specific application. Dedicated future studies are needed to explore SAVR’s potential ability to improve residual lung function.

Limitations of this study include its retrospective nature and heterogeneity of dosing regimens and imaging schedules. Our study may be underpowered to detect significant associations with lung volume reduction. Ventilation-perfusion (V/Q) scans were unavailable, but could elucidate functional changes occurring post- SABR. Analysis of pre- and post-LVRS V/Q scans do demonstrate improvements in the pathological shunting observed with severe emphysema [24]. The general consensus, however, is that the most important functional improvements after LVRS are likely the improvements in pulmonary mechanics which result directly from reductions in the lung volume. Thus, exploration of volume changes utilizing anatomical landmarks, such as lung fissures, remains a valid approach in exploring non-invasive LVRS alternatives.

For the most straightforward assessment of relative volume reduction, we analyzed lobar volume changes. However, as discussed above the volumes of high biologically effective dose were not anatomically confined within lobar boundaries. As such, our analysis would be expected to underestimate both the degree of lung volume reduction within the irradiated lung volume as well as the compensatory volume expansion of the surrounding unirradiated lung tissue, and our estimates of effect size may be considered conservative.

A prospective study of SABR to achieve volume reduction in emphysema is warranted and is ongoing at our institution for patients with severe emphysema and excessive surgical risk, in which we target localized emphysematous lung parenchyma with an ablative SABR-like dose to the center of the target region while achieving a V_60BED3_ of about 10 % of TLV in the dose gradient outside the high-dose region receiving 45 Gy in 3 fractions.

## Conclusions

In conclusion, our study reported here demonstrates further proof of principle that the lung volume reduction observed following SABR for lung tumors may provide an alternative to LVRS for non-surgical candidates. Additionally, our findings delineate the time course and factors affecting the magnitude of volume changes post-SABR. These data further inform our prospective trial utilizing SAVR for severe emphysema in poor candidates for LVRS.

## Additional file

Additional file 1: Table S1. Exploratory testing of interaction between V60BED3 and pretreatment variables. (PDF 256 kb)
