# Supplementary material for: Time course and predictive factors for lung volume reduction following stereotactic ablative radiotherapy (SABR) of lung tumors
Source: Radiat Oncol. 2016 Mar 15;11:40. doi: 10.1186/s13014-016-0616-8 (PMC4791793; doi:10.1186/s13014-016-0616-8)
Supplement: Additional file 1: Table S1. — Exploratory testing of interaction between V60BED3 and pretreatment variables. (PDF 256 kb) [file 13014_2016_616_MOESM1_ESM.pdf]

Variable tested for interaction with V_{60BED3} using multivariable linear regression	Sample number	Interaction term coefficient	Coefficient 95% Confidence Interval	R^2 value	Multivariable linear regression interaction term p-value
Pretreatment FEV1%	64	0.0005	-0.01-0.07	0.55	0.92
Fractions (single vs. multiple)	70	0.14	-0.4-0.66	0.52	0.61
Location (upper vs. lower)	70	0.0004	-0.37-0.37	0.52	0.91
Proximity (central vs. peripheral)	70	0.1	-0.28-0.48	0.52	0.6
Follow-up time	70	-0.01	-0.06-0.04	0.53	0.69
Treated lobe volume<-910 HU	70	0.0002	-0.009-0.009	0.52	0.97
Age	70	-0.02	-0.04-0.001	0.54	0.06
Pretreatment WBC count	65	0.05	-0.03-0.12	0.52	0.2
Pretreatment absolute neutrophil count	58	0.08	-0.02-0.18	0.49	0.1
Pretreatment neutrophil-lymphocyte ratio	58	0.09	-0.05-0.23	0.48	0.21
V_{20BED3}-PTV ratio	70	-0.0001	-0.0005-0.0003	0.52	0.54
V_{20BED3} Shell (subtracting V_{40BED3})	70	-0.08	-0.2-0.04	0.54	0.2
Mean SUV TLV	69	-0.77	-1.71-0.17	0.53	0.11
TLV SUV85	69	-0.5	-1.16-0.16	0.53	0.14

TLV SUV90	69	-0.4	-1.0-0.20	0.53	0.18
TLV SUV95	69	-0.28	-0.8-0.22	0.52	0.27
Mean SUV Ipsilateral lung	69	-0.7	-1.57-0.18	0.53	0.12
Mean SUV Treated lobe	69	-0.54	-1.32-0.23	0.53	0.17
Maximum SUV GTV	69	0.002	-0.03-0.03	0.51	0.9
CyberKnife vs Trilogy/TrueBeam	70	0.43	-0.63-1.49	0.53	0.50
